# Supplementary material for: ICMR task force project- survey of the incidence, mortality, morbidity and socio-economic burden of snakebite in India: A study protocol
Source: PLoS One. 2022 Aug 22;17(8):e0270735. doi: 10.1371/journal.pone.0270735 (PMC9394808; doi:10.1371/journal.pone.0270735)
Supplement: S1 File — (DOCX) [file pone.0270735.s001.docx]

**PARTICIPANT INFORMATION SHEET AND CONSENT FORM:**

**Title: Nationwide Study to estimate incidence, mortality, morbidity and economic burden due to snakebite in India.**

**Study Team:**

*Principal Investigator*: Prof Jaideep C Menon, Head, Preventive Cardiology & Population Health Sciences**,** Amrita Institute of Medical Sciences & Research Centre, Amrita Vishwa Vidyapeetham, Kochi, Kerala-682041. Email: [menon7jc@gmail.com](mailto:menon7jc@gmail.com)

*Co-Principal Investigator*: Omesh K Bharti, State Epidemiological Officer, Department of Health & Family Welfare, Shimla, Himachal Pradesh-171009, India, Email: [bhartiomesh@gmail.com](mailto:bhartiomesh@gmail.com)

*Co-Investigators*

Rupinder S Dhaliwal, Scientist ‘G’ & Head, Non-communicable Diseases Division, Indian Council for Medical Research (ICMR), New Delhi-110029, India. Email: [dhaliwalrs.hq@icmr.gov.in](mailto:dhaliwalrs.hq@icmr.gov.in)

Denny John, Adjunct Assistant Professor, Department of Public Health, Amrita Institute of Medical Sciences, Amrita Vishwa Vidyapeetham, Kochi, Kerala-682041, India – 682041. Email: [djohn1976@gmail.com](mailto:djohn1976@gmail.com)

Geetha R Menon, Scientist Grade E, National Institute of Medical Statistics- ICMR, Indian Council of Medical Research, New Delhi-110029, Email: [menongr.hq@icmr.gov.in](mailto:menongr.hq@icmr.gov.in)

Joy K Chakma, Scientist E, Non-Communicable Diseases Division, Indian Council of Medical Research, New Delhi-110029, India, Email: [drjoyk@icmr.gov.in](mailto:drjoyk@icmr.gov.in)

Background: There are about 3 million snakebites in India every year leading to an estimated 50,000 deaths. Most bites occur in rural areas where proper treatment is not available. Very few studies on the epidemiological data on the prevalence, incidence, morbidity and cost of illness resulting from snakebite in India have been conducted.

Purpose: We are studying the incidence, morbidity, mortality and cost of illness resulting from snakebite spread over 9 states in India. From this we would have valuable data which would help change treatment practices in India and help in better planning and outcome of the disease.

Procedure: ASHA workers in your area on receiving information regarding death due to snakebite in her area administer the questionnaire to victims or immediate family members in case of minor victims and in cases of death. The data is captured on a questionnaire book by ASHA workers where the victim resides. Some of the information would be related to the death of your family member due to snakebite. We understand that this might be difficult to communicate with us, however the information would be useful for us so that we get advocate to the government for appropriate policy and strategies for management of snakebite in the country.

Duration: The period of our study is for 18 months. Each interview would take 20-30 minutes.

Study geography: This study is being conducted across 336 blocks from 31 districts covering a total population of approximately 83.9 million will be included in the study. The detailed list of the study geography is mentioned in Page 3 of this document.

Study data: The questionnaire administered will capture details of victim, profile of envenomation and complications thereof, other related characteristics, treatment outcome and any other related details, and medical attention seeking behaviour pattern among the bitten (preference for modern vis-à-vis alternate systems of medicine).

Benefits: You may or may not benefit from the study, however, we believe the study would help promote betterment of the cause of snakebite victims and this will serve as a model for roll out in other parts of the state and country as well.

Risks: The collection of data on snakebite from victims is not expected to cause risks to your health or to your healthcare.

Withdrawal: You are free to withdraw from the study at any stage and take back consent for use of your data.

Compensation: As the healthcare workers will collect information at your convenient place, there will be no compensation paid for participation. Although no injury is expected, in case of unforeseen research related injury appropriate care would be provided.

Confidentiality of data: Your data will be in electronic, anonymised format and held in a secure, password-protected, encrypted environment. The data will not be used for any other purpose than this research and will not be disclosed to any third parties. The data will be held locally and only the researchers named above will be able to access it. However, this data would be handed over to the Govt Health authorities as required and used for publications in Medical journals without identifying you.

Contact details: If you have any query related to this study you may contact me ((Dr Jaideep C Menon 9447352684 the PI and if you have any complaints you may contact (Dr Shantikumar Nair, member secretary , EC AIMS at 04842801234).

INFORMED CONSENT FORM

I certify that I have understood the details about the study including risk, benefits, confidentiality and compensation and my questions have been addressed. I understand that I can withdraw from the study anytime during the period of the study. I voluntarily consent for use of my healthcare data to be used in this research study subject to the above stipulations.

………………………………………. ……………………………………………

Name of participant Signature/thumb impression and Date

………………………………………. ……………………………………………

Name of LAR Signature/thumb impression and Date

………………………………………. ……………………………………………

Name of impartial witness Signature and Date

**Table 1: Included districts, population and topography**

| Zone | State | Disricts | Taluks | Population | Topography |
| --- | --- | --- | --- | --- | --- |
| West | Maharashtra | Raigad | 15 | 2,966,109 | Coastal |
|  |  | Pune | 13 | 10,617,513 | Highland |
|  |  | Nanded | 16 | 3,784,815 | Semi-arid Deccan plateau |
| Central | Rajasthan | Jaisalmer | 3 | 756,681 | Dry arid desert |
|  |  | Udaipur | 11 | 34,55,041 | Highland |
|  |  | Bikaner | 8 | 2,363,937 | Predominantly desert |
| South | Kerala | Ernakulam | 15 | 3,695,969 | Western Ghat section |
|  |  | Kannur | 7 | 2,840,901 | Western Ghat section |
|  | Tamilnadu | Tiruchirapalli | 14 | 3,065,298 | Predominantly Highland |
|  |  | Tiravanur | 14 | 4,197,845 | Coastal |
|  | Andhra Pradesh | Nellore | 5 | 3,336,965 | Coastal |
|  |  | Chitoor | 3 | 4,699,996 | Hilly Highland |
| East | Odisha | Cuttack | 25 | 2,955,153 | Coastal |
|  |  | Sambalpur | 20 | 1,172,277 | Highland |
|  | West Bengal | Bankura | 22 | 4,049,855 | Forest and Highland |
|  |  | East Burdwan | 31 | 8,696,844 | Riverine plain |
|  |  | Jalpaiguri | 7 | 4,360,824 | Sub-himalayan Terai |
|  |  | South 24 Paraganas | 5 | 9,190,368 | Mangrove forest |
| North | Himachal Pradesh | Kangra | 15 | 1,700,344 | Sub-Himalayan plains, lowlands, and highlands |
|  |  | Una | 5 | 586,841 | Sub-Himalayan plains, lowlands, and highlands |
|  |  | Chhamba | 7 | 584,484 | Sub-Himalayan plains, lowlands, and highlands |
|  | Uttarakhand | Nainital | 8 | 1,074,885 | Hilly and forest |
| North-East | Arunachal Pradesh | Papum Pare | 15 | 1,98,821 | Hilly and forest |
|  |  | Pakke Kessang | 5 | 88,605 | Hilly and forest |
|  | Meghalaya | East Khasi Hills | 11 | 929,988 | Hilly and forest |
|  |  | West Garo Hills | 7 | 7,24,346 | Hilly and forest |
|  | Mizoram | Aizawl | 5 | 400,309 | Hilly and forest |
|  |  | Lunglei | 4 | 161,428 | Hilly and forest |
|  |  | Champai | 4 | 125,745 | Hilly and forest |
|  | Tripura | Dhalai | 8 | 425,887 | Hilly and forest |
|  |  | South Tripura | 8 | 986,377 | Hilly and forest |
| Total | 13 | 31 | 336 | 83,977,167 |  |
