# Supplementary material for: ICMR task force project- survey of the incidence, mortality, morbidity and socio-economic burden of snakebite in India: A study protocol
Source: PLoS One. 2022 Aug 22;17(8):e0270735. doi: 10.1371/journal.pone.0270735 (PMC9394808; doi:10.1371/journal.pone.0270735)
Supplement: S2 File — (DOCX) [file pone.0270735.s002.docx]

**Annexure 2: Study questionnaire**

| **GENERAL INFORMATION** | | | | | | | | | | | |
| --- | --- | --- | --- | --- | --- | --- | --- | --- | --- | --- | --- |
| 1 | Unique code (leave blank) | | | | |  | | | | | |
| 2 | Name of the patient (Write full name) | | | | |  | | | | | |
| 3 | Age (years) | | | | |  | | | | | |
| 4 | Gender | | | | | 1.Male | | | | 2.Female |  |
| 5 | Address(District / Panchayat/Village/ Town/City/State) | | | | |  | | | | | |
| 6 | Occupation | | | | | 1. Home maker  2. Farming  3. Skilled labor  4. Unskilled labor  5. Business  6. Service sector  7. Others | | | | | |
|  | DETAILS OF THE SNAKEBITE | | | | |  | | | | | |
| 7 | Date of bite | | | | |  | | | | | |
| 8 | Time of bite | | | | |  | | | | | |
| 9 | Any previous history of bite | | | | |  | | | | | |
| 10 | What was the victim doing at thetime of the bite? | | | | | 1 Walking  2 Sleeping  3 While at work  4 In the filed / plantation  5 Inside the house  6 Tending to live-stock  7 Others | | | | | |
| 11 | Geographical location where thevictim was bitten | | | | | 1. House  2. Road  3. School  4. Field/agricultural land  5. Playground  6. Other(specify) | | | | | |
| 12 | Site of bite | | | | |  | | | | | |
| 13 | Immediate first aid –Tourniquet/pressure bandage | | | | | Yes No | | | | | |
|  | DETAILS OF SNAKE | | | | |  | | | | | |
| 14 | Type of snake | | | | | Non-venomous/ Hematotoxic/Neurotoxic/Sea snake / Dry | | | | | |
| 15 | Species | | | | | Russels Viper/Cobra/Krait/Saw Scaled viper/Sea snakes/Other venomous species | | | | | |
| 16. | Snake capture | | | | | Caught/Killed | | | | | |
| **HOSPITALIZATION DETAILS** | | | | | | | | | | | |
| 17. | Was the victim hospitalized? | | | | | | Yes No | | | | |
| 18. | Name of the Hospital | | | | | |  | | | | |
| 19. | Date and time of admission | | | | | |  | | | | |
| 20. | Type of facility | | | | | | Alternate treatment/PHC/Taluk Hospital/DistrictHospital/ Medical college/Private hospital | | | | |
| 21 | If treated with alternate treatment  Date and time of presentation | | | | | |  | | | | |
| 22 | How was the victim transported to hospital | | | | | | 1. Ambulance 2. Private vehicle 3. Taxi / Autorickshaw 4. Scooter / Bike 5. Public transport 6. Others | | | | |
| 23 | Localized reaction at bite site | | | | | | Yes No | | | | |
| 24 | Vials of ASV used | | | | | | Yes No | | | | |
| 25 | Adverse reaction to ASV | | | | | | Yes No | | | | |
| 26 | If ASV discontinued due to adverse reactions | | | | | | Yes No | | | | |
| 27 (a) | Neurological symptoms present | | | | | | Yes No | | | | |
| (b) | If yes, what was the symptom | | | | | | Ptosis  Muscular paralysis  Respiratory paralysis  Numbness and tingling  Peripheral neuropathy  Others | | | | |
| 28 (a) | Bleeding manifestations present | | | | | | Yes No | | | | |
| (b) | Site of Bleeding | | | | | | Mouth and oral cavity  Airways and Lungs  Gastrointestinal  Hamaturia  Bite site / cannula  Ears  Uterine or vaginal  Intra-muscular  Intra-cranial  Others | | | | |
|  |  | | | | | |  | | | | |
| 29 | Use of blood products | | | | | | Yes No | | | | |
| 30 | Requirement of Dialysis | | | | | | Yes No | | | | |
| 31 | Requirement of ventilator | | | | | | Yes No | | | | |
| 32 | Requirement for wound debridement | | | | | | Yes No | | | | |
| 33 | Date of discharge | | | | | |  | | | | |
| 34 | Co-morbidities present | | | | | | Yes No | | | | |
| 35 | What were the Co-morbidities mentioned? | | 1.Diabetes  3. Dyslipidemia  5. CVD | | | | 2.Hypertension 4. COPD  6. Others(Pl specify) | | | | |
| 36 | | Any long term sequlae of the bite | | | 1. Psychological  2. Amputations/ Disabilities 3.Skin lesions(eczema etc)at bite si te  4. Neurological sequel  5. Cardiac sequel  6. Endocrinalabnormalities  7. Renal complications  8. Any other | | | | | | |
| 37 | | Patient died | Yes/No | | | | | | | | |
| 38 | | Date of death |  | | | | | | | | |
| 39 | | Where did he die? | Home Hospital | | | | | | | | |
| 40 | | Any other details |  | | | | | | | | |
| **ECONOMIC DETAILS** | | | | | | | | | | | |
|  | | **WAGE LOSS** | | **Patient** | | | | | **Care- giver** | | |
| 41 | | Number of days of wage loss | |  | | | | |  | | |
| 42 | | Amount per day | |  | | | | |  | | |
| 43 | | Amount paid out of pocket for  hospitalization | |  | | | | |  | | |
| 44 | | Any distress borrowing | | Yes No | | | | | | | |
| 45 | | Any collateral mortgaged, if so  what | |  | | | | | | | |
| 46 | | Was the patient covered under  any scheme like Jan Aarogya/Insurance for hospitalization? | | Yes No | | | | | | | |
| 47 | | If Yes, type of Insurance | | 1. Self - Public   -(Jan Arogya)--------------------   1. Self - Private   _____________   1. Self employer provided   (ESI)  ____________   1. State Insurance 2. Covered by family plan of children   _____________   1. Others | | | |  | | | |
| 48 | | Reimbursement amount(INR) | |  | | | | | | | |
| 49 | | Any disability insurance | | Yes No | | | | | | | |
| 50 | | Amount (INR) | |  | | | | | | | |
| 51 | | Any other financial support (detail) | | Yes No | | | | | | | |
| 52 | | Amount (INR) | |  | | | | | | | |
| 53 | | Are you a BPL card holder | | Yes No | | | | | | | |
| 54 | | Total number of earning members in family | |  | | | | | | | |
| 55 | | Monthly income of family (INR) | |  | | | | | | | |
| 56 | | Totalnumberoffamilymembers | |  | | | | | | | |
| 57 | | For patient not living with family or living with co-workers | |  | | | | | | | |
| 58 | | Monthly income of patient (INR) | |  | | | | | | | |
| 59 | | Life Insurance | | Yes No | | | | | | | |
| 60 | | Amount (INR) | |  | | | | | | | |
