# Supplementary material for: ICMR task force project- survey of the incidence, mortality, morbidity and socio-economic burden of snakebite in India: A study protocol
Source: PLoS One. 2022 Aug 22;17(8):e0270735. doi: 10.1371/journal.pone.0270735 (PMC9394808; doi:10.1371/journal.pone.0270735)
Supplement: Attachment — Submitted filename: Rebuttal_Reviewers.docx [file pone.0270735.s003.docx]

**REBUTTAL TO THE COMMENTS OF THE REVIEWERS**

**Additional Editor’s comments-**

1. We have modified the manuscript to the journals requirements and hope the revised draft reads better

2. As mentioned in the Introduction, prior studies suggest that only 20-30% of individuals of snakebite visit a modern medicine practitioner while the rest mostly depend on alternate cures available readily in most villages. Roughly two-thirds of bites are non-venomous and half the rest from venomous species are “dry” (no envenoming), which lends credibility to the alternate cures as only about 15-20% of all snakebites lead to envenoming. The basis of planning this as a community based study was so as to be able to assess numbers from the community itself and thereby be able to capture details of snakebites treated by alternate systems as well.

As mentioned details of envenoming and sequel would be captured in all cases irrespective of whether hospitalised or not as given in the Questionnaire sheet.

3. There has not been a study on the incidence and socio-economic burden of snakebite in India the only data available on a pan-India basis (1-MDS) is mortality data only (1-MDS study covered 13 states and not the whole country). The pattern of injuries would be as per the major complications and sequel as generally encountered with venomous snakebite which we think has been adequately addressed. The loss of wages is calculated for both the victim and the care-giver. Cost of treatment calculation used is the same for both fatal and non-fatal victims.

4. As mentioned this is a community based study and course in hospital and treatment may not be uniformly available. What would be entered is the number of vials of ASV used and treatment of complications vis a vis dialysis, ventilator support, use of blood products etc. Q-19 includes Alternate treatment and tells us about the treatment behaviour. As per the suggestion of the reviewer a additional Question has been added to the appended Questionnaire sheet (Date and time of the victim presenting to the Alternate treatment source).

5 a. As mentioned in the section Data collection, monitoring and checks. Identification of victims of snakebite at the ward level would be by ASHAs of the ward and the interview is to be conducted by the Field Officers. One of the exclusion criteria is non-ophid bites and snakebite would be ascertained from the interview of victims, symptoms, fang marks etc and records in case admitted, the same applying to cases of death.

b. Has been corrected to two weeks of bite or discharge from hospital / death.

c. ASHAs identify the victims for field-officers to conduct the interview.

d. The field-officers are employed specifically for the study recruiting as per ICMR norms for field-officers. Training is imparted on the data entry, common questions asked, symptoms, common venomous snakes, preventive and first-aid measures to be followed in case of bite. A half day training session is done for the field officers of the district with a follow up training after 2 weeks. The same has been done for the states of Rajasthan and Kerala. This is alongside sessions for ASHA workers of the district on the common snakes, signs and symptoms etc. The ASHA training is at the Block / Taluk level and hence multiple sessions for ASHAs are required for a single district. These sessions are in the vernacular with the questionnaire sheets, consent sheets etc being in the local vernacular.

e. The Questionnaire and Consent sheets are in the local vernacular and have been forward and backward translated by linguists.

6. ASHAs identify victims of snakebite in their respective wards of the study districts, followed by interviews of the victims by the Field Officer in a Printed Questionnaire sheet which is in the vernacular, the entered details are then entered in English on the Tab provided to the district coordinator for the same which gets stored on the server in AIMS, Kochi the nodal centre for the study. The fields for entry on the Tab PC are the same as given in the Questionnaire other than for being in English.

7. The likelihood of recall bias in those envenomed in unlikely but likely in non-venomous bites.

8. Thank you, the same has been corrected and affiliations added

b. the wording has been changed to read, there is not adequate data on the incidence and socioeconomic burden of snakebite on a national level in India.

c. I agree with the reviewer in that the Questionnaire does not empathise with the family of the victim in case of death, the field officers/ASHAs will be trained to educate the family/community so as to help prevent further incidents with simple easy measures. Necessary changes have been mada.

d. ASHAs being residents of the ward that they represent typical 1 ASHA for a 1000 population are generally aware of the health status both chronic and emergent conditions of residents of the ward that they serve.

e. Thank you the same has been corrected.

f. The same has been corrected

g. Thank you the same has been incorporated.

9. Budget has been removed

**Reviewer 2 #**

As a reply to the major comments:

The proposed study is a community based study in which victims of snakebite are identified by frontline health workers (FLHW) in their respective wards. Wards are the smallest administrative sub-units of a village (panchayat), town or corporation. Each ward elects a member to the village / town council, likewise each ward has a frontline health worker (ASHA- Accredited Social Health Activist) as representative for the health sector at the ward level. On an average there is 1 ASHA to a population of 1000. FLHWs work as the eyes and ears of the health system with an ASHA to a ward (smallest administrative subunit of the village or town). The duties of ASHAs include and not limited to immunisation and family planning, facilitating directly observed therapy short-course (DOTS) therapy for tuberculosis at ward level, surveillance and monitoring for communicable diseases- polio, vector borne febrile illnesses etc, surveillance for NCDs at the ward level, sanitation related activity, participation and facilitation of state or district specific activities at the community level.

In the present study we use FLHW to identify victims who are then interviewed by the field officer. The interview is entered on the Questionnaire sheet in the vernacular (copy in English –Annexure 2). The gathered details are then entered in English on Tab PCs by the district coordinator which gets synced to the server.

As mentioned this being a community based study and given that FLHWs are well aware of the health incidences in their respective wards we are confident most incidences would be captured.

As hospitalisation is required for administration of ASV discharge summaries / death summaries of the victims are used for the treatment details including complications/ duration of stay/ vials of ASV etc.

We agree to the reviewer’s comments that the taluks and districts were not randomly selected and cannot be generalised to the country as a whole, but the districts have been picked so as to be representative of the state.

Minor : ASHAs, AIMS etc have been expanded, the same has been corrected, thank you

**Reviewer 3 #**

Thank you for your comments

We have modified the manuscript as per your suggestions, considerably shortening the Introduction. We have detailed in the Methods section about collection of data from at the community level using Frontline health worker for the same. Treatment data would be as gathered from the discharge / death summary.

The section on the Big 4 and relevant portions have been omitted all together.

The data collection section highlighting the role of ASHA for the present proposal and their other duties have also been re-written. The district coordinators and PIs of the district are tasked with verification of entered data by way of phone calls. Random verification of data entered and gathered through the ASHA workers and field officers would be done by the District Coordinator and PI of the district.
