# Supplementary material for: ICMR task force project- survey of the incidence, mortality, morbidity and socio-economic burden of snakebite in India: A study protocol
Source: PLoS One. 2022 Aug 22;17(8):e0270735. doi: 10.1371/journal.pone.0270735 (PMC9394808; doi:10.1371/journal.pone.0270735)
Supplement: Attachment — Submitted filename: Response to reviewers.docx [file pone.0270735.s004.docx]

Reviewer #3: This new version of the protocol is more precise and concise, showing the procedure for a massive effort to gather information on the snakebite in India from the perception of those affected. I believe that the modifications made by the authors in this new version are adequate, and the protocol can be published.

Thank you for your comment.

Reviewer #4: Thank you very much for inviting me to review this important study protocol to study the incidence, mortality, morbidity and socio-economic burden of snakebite in India. Overall I would like to congratulate the authors on developing a comprehensive protocol. However, there are few major concerns, especially with regard to the sampling strategy and data collection, which need to be addressed by the authors for the data collected to be accurate and reflective of the actual burden of snakebites in the population studied.

Study aims

Should be corrected as ‘Our study aims to estimate the epidemiological and socio-economic effects due to snakebite in India’

Response: Thank you, the same has been corrected.

Study design

The authors need to give more details of the sampling strategy including how the individual blocks are selected within each districts, how many wards are roughly included in a block etc. It would be clearer if the authors could use a flow diagram to show the sampling strategy.

Response: The following text has now been inserted in Study Design section

The cross-sectional population in this case is the study population comprising all resident individuals in the study district.

Data collection

There major concerns with regard to the adequacy and accuracy of the data collection.

1. The authors state that the ASHAs will cover each ward that will include 1000 population. Is there a possibility that ASHAs may miss some of the snakebites that occur? If so what are the measures the investigators have taken to minimize missed reporting? Have they thought of carrying out public awareness campaigns about informing about the snakebites to their ASHAs before the study is started?

Response: Following text has now been inserted in the manuscript.

We are aware that there would be instances where ASHAs would might miss some of the snakebites that occur. These will the minimized to a large extent through the following; training sessions for ASHAs for identification of any warning symptoms in case of a bite, do’s and dont’s following a bite, and identification of the common venomous snakes of the region. These training sessions will also include use of Google Forms using tablets for ASHAs to enter the data as mentioned in Annexure 2. Additionally, through the grant, we have also kept a provision for incentivising the ASHAs for reporting each snakebite case to the District Coordinator.

2. The authors need to provide more information on the gathering of information and filling the questionnaires by the field workers. It appears that most of the information will be obtained through hospital discharge notes. What happens if there are no or incomplete discharge summaries? How are the investigators going to obtain information about individuals who have sought native/ local treatment where there will be no treatment records?

Response: Following text has now been inserted into the manuscript.

In cases where the discharge/death summaries are incomplete, the District Coordinator will be informed who will coordinate with the Principal Investigator of the state to reach out to the hospital/doctor to receive all relevant details. For the cases where the victim has sought native or local treatment, this will be documented by the ASHAs based on discussions with the victim, and will be supplemented by any additional information by the District Coordinator wherever needed.

3. One of the main concerns of the study protocol is the accurate identification of the snake species. They need to provide more details on how this will be done. Are they going to rely on hospital records for this? Are they going to limit species identification to cases where the dead or the live snake was brought to the hospital? Will they include species identified based on the description of the snake?

Response: This study is not designed to identify the biting species. The drop down menu in the questionnaire sheet would show a broad division into hematoxic and neurotoxic based on symptoms / discharge records (in those admitted). We have given a provision for entering the photograph of the snake if available. The study is basically designed for the incidence, mortality, morbidity and not as much for the identification of the snake species.

Reviewer #5: Good study proposal on an epidemiological topic that appears to have a dearth of current literature. Challenges that likely will arise include lack of confirmation of snake, recall bias, dry bites, etc. Would recommend keeping working data evaluating whether the snake was visualized, how the confirmation took place (photo, visual, combined with consistent clinical findings) to get a better sense of distribution. This could also help inform the geographic distribution patterns of specific species in different areas. This project will be a big undertaking, however is worthwhile and ambitious. Determining socioeconomic burden may prove challenging also as metrics will need to be normalized across various income levels, etc. May be worthwhile tabulating total costs of treatment as an objective figure to get a sense of the baseline findings. Are the median income levels and socioeconomic determinants of each geographical area/district being considered? Overall, this is an interesting study and I look forward to the findings.

Response: Thank you for your comments. This study is not designed to identify the biting species. The drop down in the questionnaire sheet would show a broad division into hematoxic and neurotoxic based on symptoms / discharge records (in those admitted). We have also given a provision for entering the photograph of the snake if available. The study is basically designed for the incidence, mortality, morbidity and not as much for snake species identification.

Yes, the income of the household is being considered. There is a provision for upload of the bitten part / snake if available. All valuable suggestions which have been considered. The other social determinants like educational background will also be considered.

Reviewer #6:

- important study but I had a few comments/concerns:

- the study design - is it prospective, a survey, or retrospective? It is said to be prospective, but recall bias is mentioned which would be an issue for a retrospective design?

Response: This is a prospective cross-sectional study study on which we look at an event of snakebite (outcome) in the cohort of a defined population (i.e. the entire population of the district) reporting incidence of snakebite. The data is to be reported within 2 weeks and the mention of recall bias was in relation to victims forgetting about the incident especially in cases of dry/ non-venomous bites, which is why the window was kept at 2 weeks.

- there is use of “his” in referring to investigators but “her” when referring to the site workers (ASHWs) - this needs to be changed to gender neutral language (eg their)
- acronyms need to be spelt out in full at first time of use - eg ASHW, NCD
- in abstract the word “ward” is used, but this is an ambiguous term - in some countries a “ward” in an inpatient unit, and so I originally interpreted this as inpatient recruitment only, and hence big flaws in capturing true incidence. Later on in paper it is defined as a small community village unit. Suggest abstract word is changed to a non-ambiguous term
- towards the end section there is use of the term “down trodden” poor people - I think socioeconomically disadvantaged probably a better/preferred term.

Response: Thank you, the same has been corrected and explained as suggested by the reviewer.

Thanks for invitation.

Reviewer #7: The authors present interesting data on a largely neglected area of research, i.e. envenoming epidemiology. The scarcity of data in this field necessitates that any data that can be published, should be published and, thus, I believe this study merits publication. However, there remain some major revisions to ensure the coherency and soundness of the presented data.

Response: We have now addressed al the comments by reviewers and incorporated them in the revised version. We would like thank the reviewers for their comments as we believed that it has helped make the protocol better.

General

Thank you for noting that your data will be made available. Please note that PLOS ONE’s policies on Data Availability require that all data necessary to replicate the findings of the manuscript be made available upon acceptance.
PLOS ONE strongly recommends that this data be deposited to a stable public repository such as Figshare (<http://figshare.com/>) or Dryad ([datadryad.org](http://datadryad.org/)). For a list of other PLOS recommended repositories, please visit: <http://journals.plos.org/plosone/s/data-availability#loc-recommended-repositories>.

Response: Annexures used in this protocol are available in public repository atJohn, Denny; Menon, Jaideep (2021): ICMR –Task Force Study on the incidence, mortality, morbidity and socio-economic burden of Snakebite in India: A study protocol. figshare. Dataset. https://doi.org/10.6084/m9.figshare.14072108.v1
